# The Proper Treatment of Enamel

**Published:** 1922-05

**Authors:** J. P. Carmichael


### THE PROPER TREATMENT OF ENAMEL

BY J. P. CARMICHAEL, D.D.S.

The enamel of a tooth is well named, as it is a covering of the nature of glass. When we deal with enamel, we must not lose sight of that fact that after it has been scratched by any hard substance, we have a most difficult problem to deal with in removing these scratches and restoring its natural brilliancy.

It is quite impossible to cut the enamel of teeth, although it can be shattered. To drill a hole in enamel, we use a cross-cut bur which tears its way through. This bur would tear its way through a piece of glass in the same manner. The same substances that scratch enamel will scratch glass; for instance, try pumice stone on a pane of glass, and notice the effect—it scratches the surface just as it does the enamel.

No matter how finely pumice stone is powdered, it remains a sharp, cutting, crystalline abrasive that has no polishing qualities. If you want to wear a surface away, use powdered pumice stone. To apply pumice to a tooth that has a natural brilliancy is nothing less than criminal. The first stroke of the polishing wheel, charged with powdered pumice, will instantly destroy the natural brilliancy and luster, and if the tooth is made dry, this condition is plainly seen. Not only has the luster been destroyed, but the porosity of the tooth texture has been opened, and the enamel made susceptible to stains and the adhesion of foreign matter. Untold harm is often done by using a dentifrice containing powdered pumice stone.

Tooth texture possesses a quality of viscosity making it capable of being drawn, an effect similar to the burnishing of metal. This is often noticeable on the occluding surfaces of the teeth where the enamel is worn away. The dentine will be found to be, oftentimes, as hard as the enamel itself, a result of the friction in masticating at the occluding surfaces whereby nature demonstrates that to polish and harden the enamel properly, friction is required. To polish the enamel of teeth, the important thing to remember is that the powder must be one that does not scratch the surface.

If we consider the condition found on the surface of a tooth that has been ground with a carborundum stone, or cut with a bur, it will show a broken, cracked surface. Finish this surface smooth with a dry sandpaper disk, followed by a dry friction powder, and the tooth will become glazed and hardened to such a degree that it will ward off all external influences. This treatment also provides a way of correcting the sensitive surfaces on teeth. In cavity preparation, the walls and cavity margins can be so smoothed and hardened as to entirely correct discolorations which are so commonly seen about fillings and inlays, called "leaky fillings."

To polish any substance, it is first necessary to work out the coarse scratches with a finer abrasive until the surface is perfectly smooth. A friction polish which possesses a definite quality of resistance for burnishing the surface is required. Obviously such a substance will be effective only when applied dry. It will restore the natural, sparkling brilliancy to enamel, even after it has been dulled or scratched by pumice or other harmful agents.—*Dental Summary.*

